# The Redistribution of Power: Neurocardiac Signaling, Alcohol and Gender

**DOI:** 10.1371/journal.pone.0028281

**Published:** 2011-12-02

**Authors:** Marsha E. Bates, Jennifer F. Buckman, Evgeny G. Vaschillo, Vladimir A. Fonoberov, Maria Fonoberova, Bronya Vaschillo, Eun-Young Mun, Adriana Mezić, Igor Mezić

**Affiliations:** 1 Center of Alcohol Studies, Rutgers – The State University of New Jersey, Piscataway, New Jersey, United States of America; 2 AIMdyn, Inc., Santa Barbara, California, United States of America; 3 Center for Control, Dynamical Systems and Computation, University of California Santa Barbara, Santa Barbara, California, United States of America; University of Cincinnati, United States of America

## Abstract

Human adaptability involves interconnected biological and psychological control processes that determine how successful we are in meeting internal and environmental challenges. Heart rate variability (HRV), the variability in consecutive R-wave to R-wave intervals (RRI) of the electrocardiogram, captures synergy between the brain and cardiovascular control systems that modulate adaptive responding. Here we introduce a qualitatively new dimension of adaptive change in HRV quantified as a *redistribution* of spectral power by applying the Wasserstein distance with exponent 1 metric (W_1_) to RRI spectral data. We further derived a new index, D, to specify the direction of spectral redistribution and clarify physiological interpretation. We examined gender differences in real time RRI spectral power response to alcohol, placebo and visual cue challenges. Adaptive changes were observed as changes in power of the various spectral frequency bands (i.e., standard frequency domain HRV indices) and, during both placebo and alcohol intoxication challenges, as changes in the structure (shape) of the RRI spectrum, with a redistribution towards lower frequency oscillations. The overall conclusions from the present study are that the RRI spectrum is capable of a fluid and highly flexible response, even when oscillations (and thus activity at the sinoatrial node) are pharmacologically suppressed, and that low frequency oscillations serve a crucial but less studied role in physical and mental health.

## Introduction

In fields as diverse as astrophysics and molecular biology, variability within a system has been identified as a characteristic of adaptability. Variability generally reflects modulatory processes that detect and dynamically respond to system changes. Heart rate variability is closely tuned to changes in the internal and external environment and, through the cardiovascular system's significant bidirectional communication with the brain [1], [2], [3], supports both cardiovascular health [4], [5] and stress and emotion regulation [6], [7]. To ensure that these integrated mind-body responses [8], [9], [10] occur rapidly and efficiently, heart rate variability changes on a moment-to-moment basis.

Heart rate variability (HRV) is measured as the variability in consecutive R-wave to R-wave intervals (RRI) of the electrocardiogram. It reflects the activity of multiple influences at the sinoatrial (SA) node of the heart, especially those emanating directly from the brain via the sympathetic and parasympathetic efferent pathways of the autonomic nervous system (ANS). Commonly used, standard frequency domain indices of HRV are generated from the RRI power spectrum density (PSD) [11] to reflect the *amount* of variability within the system calculated as the power of RRI oscillations in various frequency ranges. The overall amount of variability in these ranges of the RRI PSD is an obviously essential dimension of neurocardiac dynamics, but unlikely the only dimension, especially if one considers the possibility that RRI PSD dynamics can change qualitatively and non-linearly in response to perturbation [12]. Reorganization of variability (i.e., redistribution of spectral power) is another key dimension of neurocardiac dynamics, which may be captured in ways other than the standard HRV indices (e.g., change in the ratio of power in the low and high frequency ranges) designated by the Task Force [11].

In this paper, we go beyond the Task Force conceptualization of spectral reorganization by measuring *reorganization* of variability independently from *amount* of variability (i.e., disentangling the redistribution of spectral power from the power of RRI oscillations in a given frequency range). We propose that, in and of itself, spectral reorganization may reflect a fundamental mechanism of adaptability that supports changes in response to challenge even when the amount of variability is diminished, for example, by disease states [13], [14] or pharmacological challenges such as alcohol drinking [15]. This implies that adaptive neurocardiac responding might still be accomplished by strategically redistributing power to a given frequency, even when *de novo* generation of spectral power from the neural inputs at the SA node is impaired (i.e., when the amount of variability is limited or less likely to increase).

This study is the first to apply the Wasserstein distance with exponent 1 (W_1_) metric to RRI spectral data in order to quantify redistribution of RRI spectral power as a distinct dimension of real time cardiovascular reactivity. Applying W_1_ to RRI data provides a strong approach to characterizing redistributions in the temporal dynamics of the RRI PSD during challenge conditions because it is robust against noise [16], [17] and makes no a priori assumptions about linearity of the data [12]. The utility of W_1_ as a measure of dissimilarity between probability distributions [16] and histograms [17] is well-established outside the area of physiology. We computed W_1_ from individual RRI PSD in order to measure dissimilarity between power spectra elicited during a resting state compared to a challenge condition, as follows:
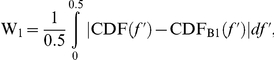
where the cumulative distribution function (CDF) is defined as
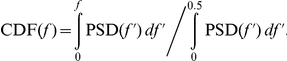



The W_1_ metric differs in several important ways from the standard indices of HRV defined by the Task Force [11], which are calculated as the absolute power in predefined spectral frequency bands (e.g., low [LF HRV] and high [HF HRV] frequency bands) or as the ratio between them (LF/HF ratio). First, the W_1_ metric integrates the RRI PSD across the entire spectral range (i.e., ∼0 to 0.5 Hz); no predefined spectral frequency bands are imposed. Second, the W_1_ is a metric on a set of equivalence classes of power spectra. In other words, the RRI PSDs being compared are normalized prior to computing W_1_. This implies that two power spectra, which can be obtained from each other by multiplying one of them by a positive constant (i.e., changes only in spectral power), will not be differentiated by W_1_. Third, the W_1_ metric measures dissimilarity between two PSDs in terms of absolute distance, and thus captures absolute change in the *overall shape or structure* of the power spectrum in response to perturbation. These features of the W_1_ metric make it possible to quantify and visualize the overall extent to which the RRI PSD reorganizes following a perturbation, distinct from increases or decreases in HRV. We suggest that a consideration of spectral power redistribution (W_1_), together with changes in power captured by the standard spectral frequency indices (e.g., HF HRV, LF HRV) may be heuristic for further understanding mechanisms that modulate neurocardiac responding.

To refine this approach for assessing change in neurocardiac signaling, it is useful to consider the direction of spectral power redistribution. A redistribution in the RRI spectrum towards lower versus higher frequencies has a different physiological meaning with respect to adaptability. There is consensus that HF HRV, dominated by oscillations around 0.3 Hz, reflects activation of the parasympathetic branch of the ANS mediated by the vagus nerve, and is commonly linked to respiration. HF HRV is thought to be dominant at rest or during restful states. In contrast, LF HRV is influenced by both sympathetic and parasympathetic activity [18] and often includes oscillations at ∼0.1 Hz (also called the Mayer wave) that are strongly mediated by the HR baroreflex system [19], [20]. Notably, 0.1 Hz is one of several resonance frequencies in the cardiovascular system [21], [22], [23] that serve to augment variability in the system [22], [24]. To capture the directionality of spectral power redistribution, we derived a new directionality index (D) from W_1_. D was computed from individual PSD (using the same CDF definition as W_1_) as follows:
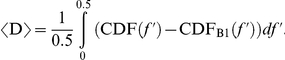
When D takes on a positive value, it suggests a coordinated reorganization towards lower frequency oscillations. Alternatively, a negative D implies movement towards higher frequency oscillations.

This paper introduces W_1_ and D as a new approach to characterizing HRV changes in response to challenge and examines whether it provides non-redundant information to standard indices of RRI spectral power (e.g., LF HRV, HF HRV, LF/HF ratio). We used existing data from a study aimed at understanding physiological mechanisms that modulate alcohol use behaviors in young men and women. The original study collected ECG data at rest and in response to alcohol, placebo, and cognitive-emotional challenges in healthy men and women, and as such provided a rich data source for assessing adaptive reactions to a tiered challenge paradigm. We chose to examine gender differences as a starting point from which to assess the contribution of spectral redistribution (W_1_) and directionality of the redistribution (D) to adaptive psychophysiological response because multiple literatures demonstrate inherent differences in adaptive functioning between men and women. For example, women have greater vagal mediation of heart rate [25] and autonomic balance [26] at rest, greater vagal withdrawal in response to stress [27], less suppression of HRV by alcohol [28], and more protection against coronary heart disease [29] compared to men. Moreover, women demonstrate a well-established survival bias which is initiated at conception [30] and evidence longer life expectancies in both developed and developing countries [31].

We utilized pharmacological (alcoholic beverage consumption) and visual cue challenges that are known to change power in the RRI PSD. Acute intoxication depresses HRV in both low and high frequency bands [15] and cognitive-emotional visual cues enhance spectral power at 0.1 Hz in the LF band [32]. We hypothesized that women would show an adaptive advantage in responding to the tiered challenge paradigm, especially as the magnitude of the challenge increased. We predicted that this adaptive advantage would be evidenced by greater spectral redistribution (W_1_) towards low frequency oscillations (D) in response to potent stimuli. A MATLAB function to compute W_1_ and D indices is available at the following URL: http://aimdyn.com/W1index/.

## Results

**Figure 1:**
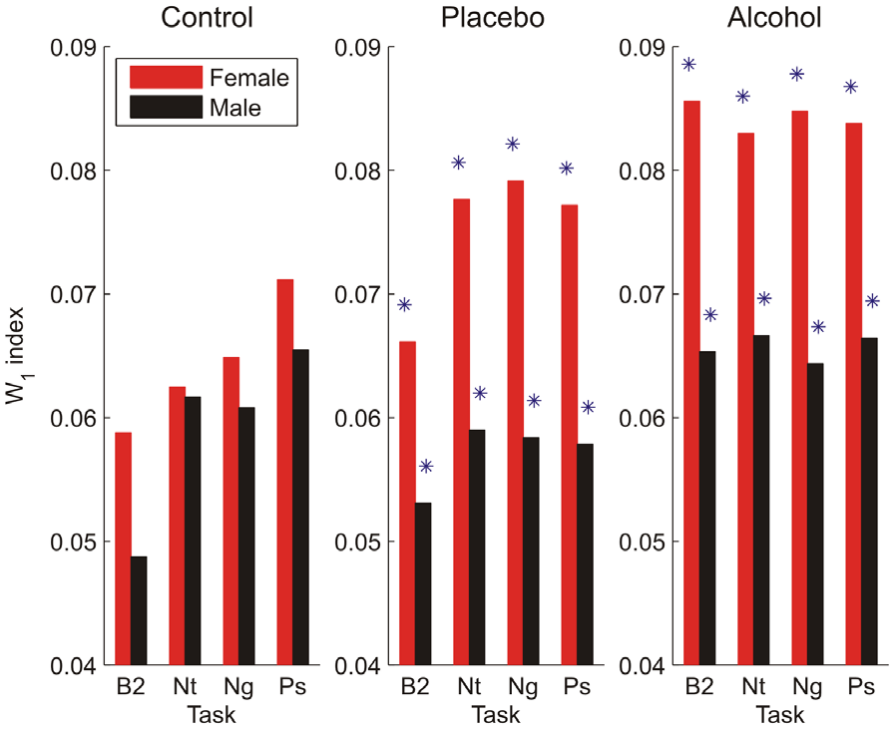
Mean W_1_ index differed for men and women. Redistribution of spectral power was observed during the control (n = 55 females, 54 males), placebo (n = 55 females, 54 males), and alcohol (n = 88 females, 84 males) beverage conditions in response to a post-drinking baseline (B2) and while viewing neutral (Nt), negative (Ng), and positive (Ps) visual cues. Women, compared to men, demonstrated a significantly greater redistribution in response to a post-drinking baseline and all visual cue stimulations during alcohol and placebo challenges (* = p<.05).

**Figure 2:**
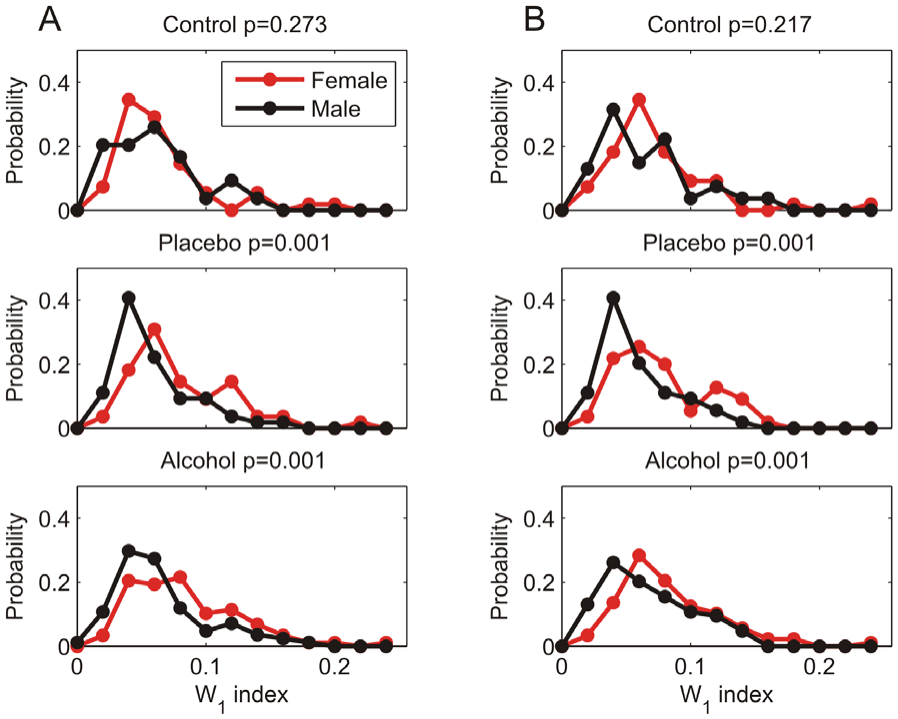
Probability distribution of W_1_ index in all subjects during the (A) negative and (B) positive visual cue tasks. There were large individual differences in spectral redistribution during all tasks. P-values indicating the significance of differences between men and women in the control (top panels), placebo (middle panels) and alcohol (bottom panels) beverage conditions are presented above each panel.

A redistribution in RRI spectral power (W_1_) was observed during all experimental challenges. Women demonstrated significantly greater redistribution of RRI spectral power in response to all visual cue stimulations during alcohol and placebo challenges compared to men (all *p*<.05, Figure 1). This gender difference was not observed in the control condition (participants were told no alcohol and received no alcohol). Figure 2 illustrates that there are large individual differences in the overall extent to which spectral power redistributes in response to the negative (Ng) and positive (Ps) visual cue challenges; however, in general, women obtain significantly larger W_1_ than men demonstrating more spectral redistribution to emotional visual cues.

**Figure 3:**
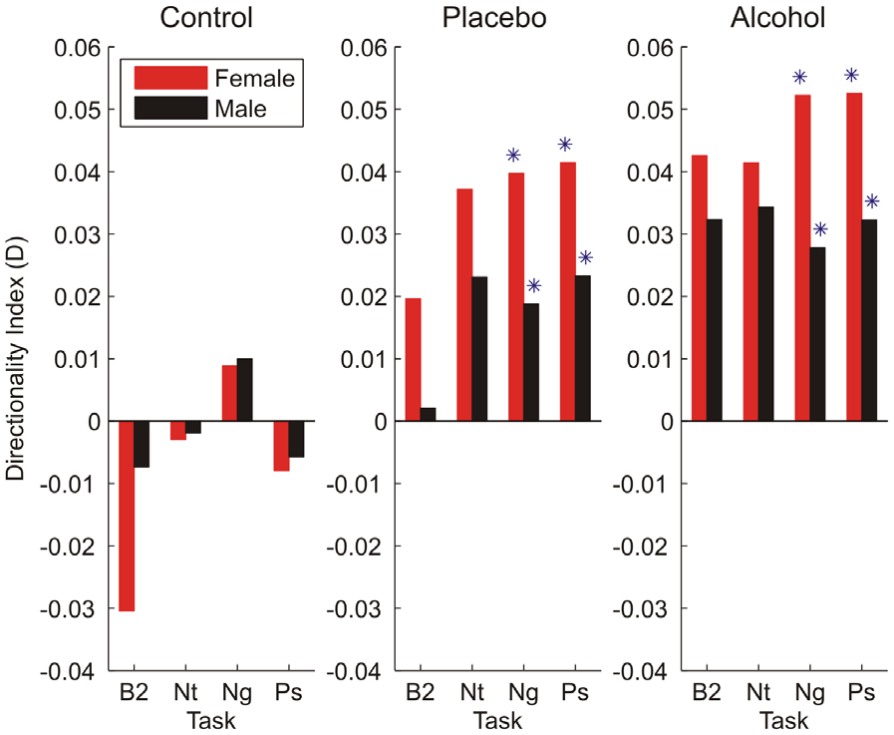
Directionality of redistribution differed between men and women. Spectral power showed little redistribution during the control beverage condition (n = 55 females, 54 males) in response to the post-drinking baseline (B2) and the neutral (Nt), negative (Ng), and positive (Ps) visual cue tasks. Spectral power redistributed towards lower frequencies in the placebo (n = 55 females, 54 males) and alcohol (n = 88 females, 84 males) beverage conditions in response to positive and negative visual cues, more so in women than men (* = p<.05).

Examination of gender differences in the D index showed that during alcohol and placebo challenges, women's RRI PSD shifted more towards lower frequencies in response to the visual cues than did men's (Figure 3). These gender differences were statistically significant for the most emotionally valenced (positive and negative) cues, but not in response to B2, nor to the emotionally-neutral cues (Nt). It is interesting to note that in the second baseline (B2) of the control condition, the averaged D values were negative (i.e., redistribution was towards higher frequencies), potentially indicating greater vagal mediation in cases where there is an absence of challenge and participants have had the opportunity to acclimate to the testing environment.

**Figure 4:**
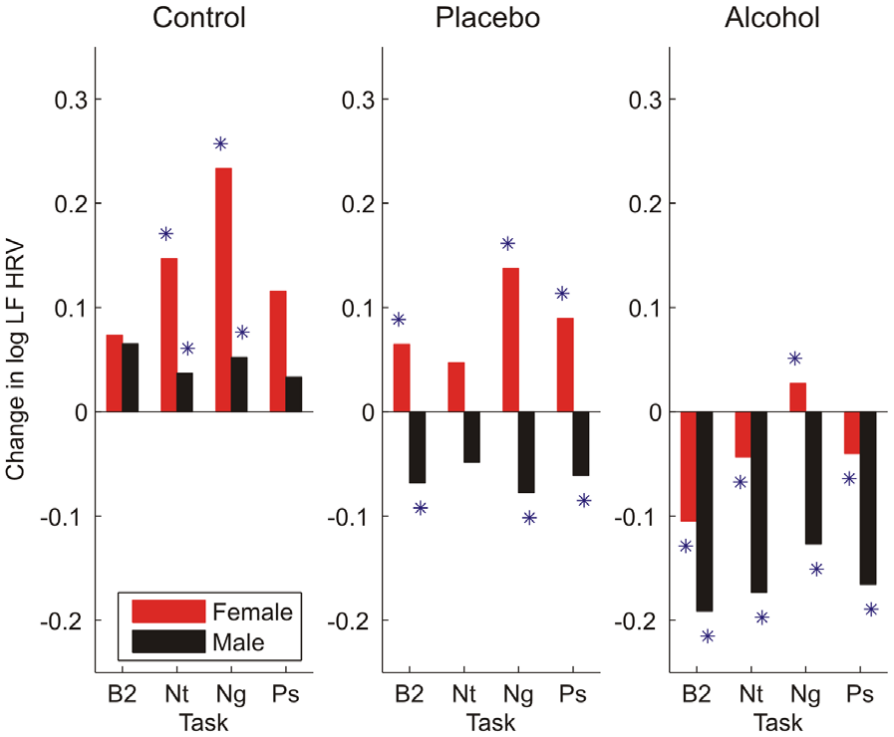
Mean change from pre-drinking baseline in the logarithm of low frequency (LF) heart rate variability (HRV) differed between men and women. Changes in LF HRV (i.e., spectral power in the LF range) were noted during the post-drinking baseline (B2) and the neutral (Nt), negative (Ng), and positive (Ps) visual cue tasks. Changes were observed in the control (n = 55 females, 54 males), placebo (n = 55 females, 54 males), and alcohol (n = 88 females, 84 males) beverage conditions (* = p<.05, men versus women).

**Figure 5:**
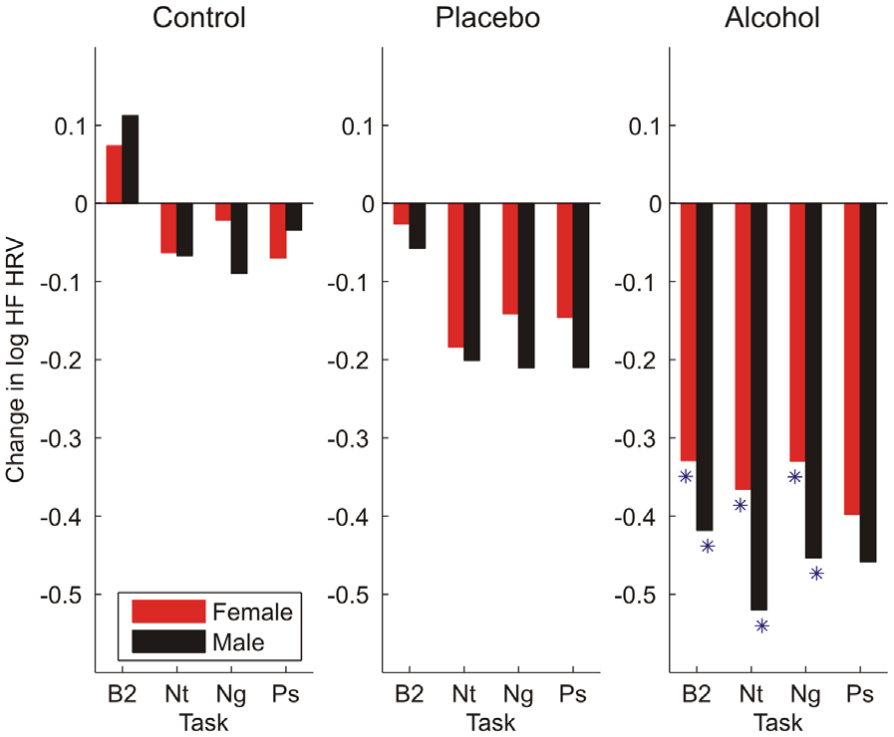
Mean change from pre-drinking baseline in the logarithm of high frequency (HF) heart rate variability (HRV) differed in men and women during intoxication. Suppression of HF HRV (i.e., spectral power in the HF range) by alcohol (n = 88 females, 84 males) was noted during the post-drinking baseline (B2), and the neutral (Nt) and negative (Ng) visual cue tasks. This suppression was generally greater in men than women (* = p<.05). No gender differences were noted in the control (n = 55 females, 54 males) or placebo (n = 55 females, 54 males) beverage conditions.

**Figure 6:**
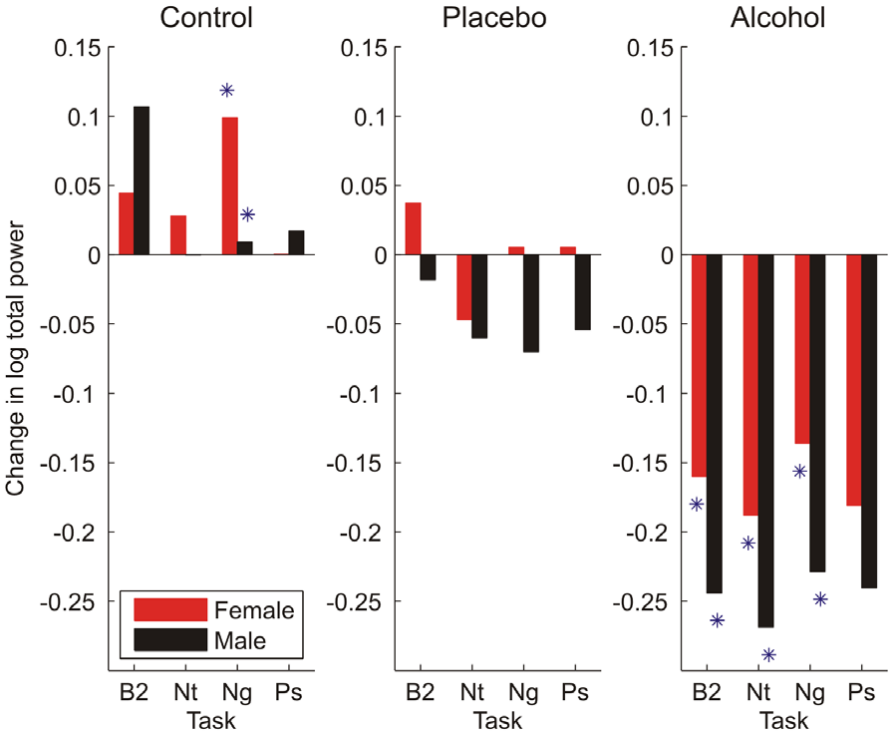
Mean change from pre-drinking baseline in the logarithm of total heart rate variability (HRV) differed in men and women during intoxication. Suppression of total spectral power by alcohol (n = 88 females, 84 males) was noted during the post-drinking baseline (B2) and the neutral (Nt), negative (Ng) visual cue tasks. This suppression was generally greater in men than women (* = p<.05). Gender differences were also noted in the control (n = 55 females, 54 males) beverage conditions in response to negative visual cues only.

**Figure 7:**
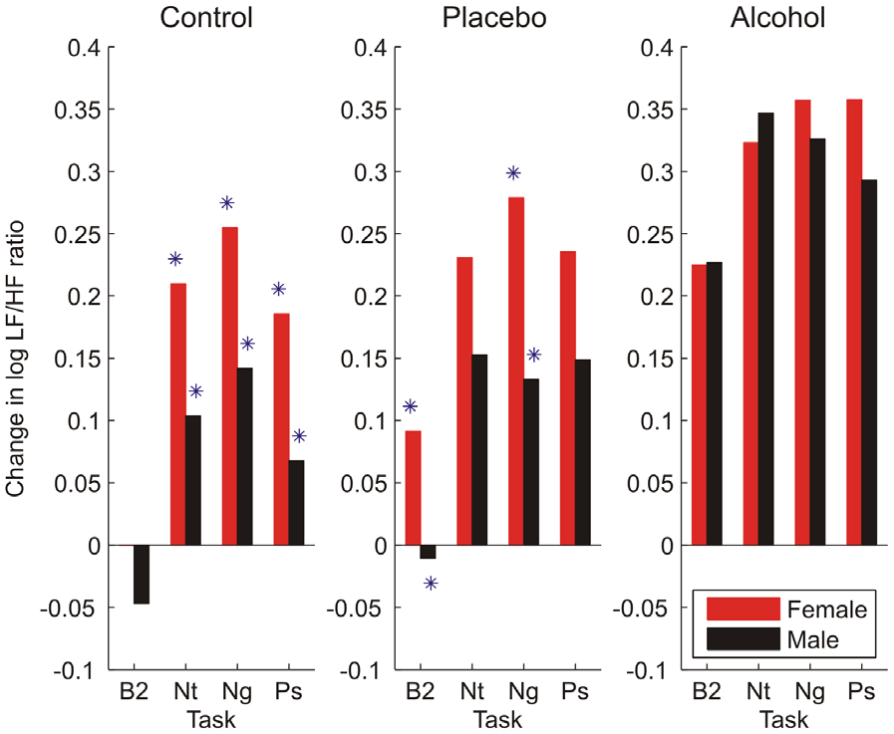
Mean change from pre-drinking baseline in the low frequency (LF)/high frequency (HF) ratio differed between men and women. Changes in the LF/HF HRV ratio were noted during the post-drinking baseline (B2) and the neutral (Nt), negative (Ng), and positive (Ps) visual cue tasks. Changes were observed in the control (n = 55 females, 54 males) and placebo (n = 55 females, 54 males) beverage conditions, but not in the alcohol condition (* = p<.05, men versus women).

Men and women also demonstrated distinct patterns of change in standard HRV indices in response to the tiered challenge paradigm. Notable changes (from baseline) in spectral power were observed in both the LF band (LF HRV) and the HF band (HF HRV) of the RRI PSD. Measured as a change from baseline, women showed greater increases in LF HRV in response to negative and neutral cue stimulation (p<.05) in the control condition (with a trend towards greater LF HRV to positive cues, p<.09). They also showed greater increases in LF HRV to all cue types in alcohol conditions (p's<.05), and to all cue types but neutral in the placebo condition (p's<.05; neutral cues p<.06, Figure 4). Gender differences in HF HRV were limited to the alcohol challenge condition, wherein women's HRV was generally less dampened by the alcohol challenge compared to men (Figure 5). These gender differences were statistically significant (p<.05) during the B2 baseline task as well as during the neutral and negative cue stimulation tasks. Changes in power in the overall RRI PSD (total HRV) also differed by gender (Figure 6). Women showed a greater increase in total HRV in response to negative emotional cues during the control condition (p<.05) and during B2 and to the neutral and negative emotional cues (p<.05) during the alcohol condition. Finally, computation of the LF/HF ratio (Figure 7) revealed a pattern of results that was distinct from the LF HRV and HF HRV indices as well as from the W_1_ and D analyses. Women had greater LF/HF ratios in response to emotion cue tasks in the control condition (p's<.05) and to the B2 and negative emotional cue task in the placebo condition (p<.05); gender differences to neutral and positive cue tasks in the placebo condition were at the level of a trend (p's<.10). No gender differences were noted in LF/HF ratio during the alcohol condition.

## Discussion

The goal of this study was to introduce a new approach for quantifying and interpreting real-time changes in the RRI power spectrum in response to challenge and new tools to assess adaptive neurocardiac changes. The main finding is that the W_1_ metric and D index are complementary to the standard HRV frequency domain indices. When increases and decreases in spectral power were considered together with the nature of the reorganization that occurred in the RRI PSD, the data revealed qualitatively different ways in which the cardiovascular system adapts to different types of challenges. The results suggest that the W_1_ metric and D index provide insight into a dimension of neurocardiac dynamics that supports adaptive responding even when neural activity at the SA node of the heart is compromised and the amount of cardiovascular variability cannot be adjusted appropriately (i.e., increase or decrease) to meet a given challenge. This is consistent with the notion that the cardiovascular system has at its disposal multiple, complementary mechanisms to support adaptive responding.

Clearly, further research and discussion are needed to understand the implications of this new dimension of spectral change; however, using gender differences as a starting point, we offer some initial speculations as to the significance of the W_1_ metric and D index that build on our ongoing research into the arterial baroreflex system and the role of resonance in adaptive responding. As a first example, in the control condition, there were no significant gender differences in spectral power redistribution (W_1_) in response to cognitive emotional visual cues, but women did show greater increases in LF HRV and a greater LF/HF ratio compared to men. We interpret these findings as showing that when the cardiovascular system, and its bidirectional communication with the brain, is performing optimally (participants were all young healthy adults), the “primary” adaptive response to the visual cue challenge was to provoke greater neural activation at the SA node to generate more low frequency oscillations; this was accomplished more robustly by women than men.

As a second example, following alcohol consumption, the power of the total RRI spectrum (total HRV index) was decreased, but both the LF HRV and HF HRV suppression was significantly less prominent in women than in men [28]. The W_1_ metric and D index further showed that the relative contribution of RRI oscillations with different frequencies to the power spectrum changed considerably, and that the extent of this redistribution was greater, and towards lower frequencies, in women than in men. One interpretation of this finding is that when neural input at the SA node is dampened by alcohol, the system attempts to compensate for this reduction by redistributing remaining power towards lower frequencies. If spectral reorganization is serving as a mechanism by which the cardiovascular system compensates for reduced neural input at the SA node and helps to offset or override the effects of alcohol, then the fact that women are both less suppressed by alcohol and more able to redistribute power towards lower frequencies is consistent with an adaptive advantage.

There is an abundance of research demonstrating that greater HF HRV at rest (i.e., unchallenged) is associated with health and well-being. Here we assert that the story is different in the face of challenge; namely, that oscillations in the low frequency range also serve important adaptogenic functions that support health and well-being. During a mild challenge, such as viewing visual cues with emotional content, an increase in power in the LF band of the RRI PSD may be adaptive because it signals activation of neural inputs to promote alertness and vigilance. When neural communication with the heart is compromised by alcohol or other adverse conditions, maintaining low frequency oscillations, even at the expense of high frequency oscillations, may be adaptive because oscillations at lower frequencies can generate a resonance response. Resonance amplifies cardiovascular oscillations and thus may compensate for reduced “efferent-induced” oscillations. We propose that through resonance, the heart-brain biofeedback loop can be maintained. It is noteworthy that both vagal stimulation [33] and resonance breathing biofeedback [34], which induces high oscillations at 0.1 Hz, show promise in the treatment of various physical and mental health problems [35], [36], [37].

As a final point, we note that our results clearly demonstrate that W_1_ does not capture the same spectral movement as the LF/HF ratio. In the control condition, a change in the shape (or structure) of the RRI PSD was detected by the standard LF/HF ratio index but not the W_1_ metric. In the alcohol condition, on the other hand, a change in the structure of the RRI PSD was detected by the W_1_ metric, but not the LF/HF ratio index. A major difference between the LF/HF ratio index and W_1_ metric is that the latter compares normalized spectra and thus can differentiate amount of variability (i.e., RRI spectral power) from organization of variability (i.e., shape of RRI PSD). Another key difference is that the ratio compares two portions of a single RRI PSD using predefined frequency bands, whereas the metric captures shifts across the entire spectral range between two independent RRI PSDs. The conceptual significance of these differences is open to debate.

In today's world, adaptation most often entails successful negotiation of a wide variety of sublethal physical and emotional challenges that seldom demand an extreme defensive response. Thus, useful conceptualization of body-brain communication needs to encompass more than “fight or flight” response capacity and focus on the nuanced biofeedback that occurs between the heart and brain on a moment-to-moment basis. As has been well studied, the human heart must receive detailed instructions from the brain to adapt rapidly and effectively to an enormous range of challenges that vary in magnitude, content, and salience. Less often considered, but also of seemingly critical importance, is the communication of information back to the brain regarding how well the body has carried out neural commands. It is necessary to move beyond the existing standard indices of HRV to initiate exploration of how such “body to brain” mechanisms participate in the closed-loop circuit of communication that continually exists between these two most essential organs. The W_1_ metric and D index may serve as an initial step towards this by helping to understand autonomic function as an embedded component of central nervous system functioning.

## Methods

### Ethics statement

This study was approved by the Rutgers University Institutional Review Board for the Protection of Human Subjects Involved in Research and participants provided written informed consent.

### Participants

The present analyses used data from 232 (118 women) young adults between the ages of 21 and 24 (mean = 21.6; *SD* = 0.9) who regularly drank alcohol, but who were not alcohol dependent. Individuals participated in a total of 390 sessions (172 alcohol, 109 placebo, and 109 control sessions). Exclusion criteria included a history of psychiatric or medical conditions that contraindicated alcohol administration or confounded interpretation of HRV, regular use of illicit or prescribed drugs, and pregnancy or lactation. The majority of participants were non-Hispanic White (63%); 18% were Asian, 11% African American, and 8% mixed or other. Participants were asked to refrain from alcohol and illicit use of drugs for the 24 hours prior to the laboratory session and eat a light, low-fat meal 3–4 hours prior to arrival. A breath sample confirmed zero blood alcohol concentration (BAC) at the start of testing.

### Procedures

After providing written informed consent, individuals completed 2 experimental sessions in which they were randomly assigned to 2 of 3 beverage conditions (alcohol, placebo, no alcohol). The participant was seated in a comfortable chair in front of a TV screen in a sound-attenuated, dimly-lit room and physiological sensors and electrodes were attached. Those in the alcohol condition were administered 3 mixed drinks that contained alcohol (0.90 ml/kg for men, 0.78 ml/kg for women) and a mixer (orange juice, cranberry juice, lime juice) in a ratio of 4 parts mixer to 1 part 95% ethanol to achieve a target BAC of .09%. Each drink was consumed during a consecutive 5-minute interval. Participants in the placebo condition consumed three equivalently volume-controlled drinks that contained mixer with 100 µl ethanol float per each cup and alcohol olfactory cues. Participants in the control condition received three volume-controlled drinks that contained 100% mixer (told no alcohol and received no alcohol). The mean BAC levels at the start and end of the cue presentation in the alcohol condition were 0.08% (*SD* = 0.01) and 0.07% (*SD* = 0.01), respectively.

Prior to beverage consumption, participates performed a 5-minute low-demand task [38] to equate cognitive load across participants (pre-drinking baseline; B1). Beverages were then consumed, and when a BAC of ∼.06% was reached (or after 5–10 min in placebo and control sessions), participants again performed the low-demand task (post-drinking baseline; B2). Participants then viewed 5-minute blocks of emotionally-valenced (negative, positive, neutral) visual stimuli (pictures [39] or words [40], [41]) in random order. Cue presentation order (15 cues, each presented twice) was randomized within each block. Each cue was presented for 5 seconds with a 5-second inter-cue interval (0.1-Hz presentation). Electrocardiogram (ECG) recordings were continuously collected (1,000 samples per second) using a Powerlab Acquisition System (ADInstruments, Colorado Springs, CO). Beat-to-beat intervals of the heart or R wave-to-R wave intervals of the ECG were measured using WinCPRS software program (Absolute Alien Oy, Finland). T-tests were used to compute statistical differences between males and females.

## References

[1] Benarroch EE (1997). Central autonomic network: functional organization and clinical correlations.

[2] Benarroch EE (2008). The arterial baroreflex: functional organization and involvement in neurologic disease.. Neurology.

[3] Goldstein DS (2001). The autonomic nervous system in health and disease.

[4] Abboud FM (2010). The Walter B. Cannon Memorial Award Lecture, 2009. Physiology in perspective: The wisdom of the body. In search of autonomic balance: the good, the bad, and the ugly.. Am J Physiol Regul Integr Comp Physiol.

[5] Thayer JF, Lane RD (2007). The role of vagal function in the risk for cardiovascular disease and mortality.. Biological Psychology.

[6] Tolkunov D, Rubin D, Mujica-Parodi L (2010). Power spectrum scale invariance quantifies limbic dysregulation in trait anxious adults using fMRI: adapting methods optimized for characterizing autonomic dysregulation to neural dynamic time series.. Neuroimage.

[7] Thayer JF, Lane RD (2000). A model of neurovisceral integration in emotion regulation and dysregulation.. Journal of Affective Disorders.

[8] Porges SW (2009). The polyvagal theory: new insights into adaptive reactions of the autonomic nervous system.. Cleveland Clinic Journal of Medicine.

[9] VasilevskiǏ NN, Sidorov YA, Suvorov NB (1993). Role of biorhythmologic processes in adaptation mechanisms and correction of regulatory dysfunctions.. Fiziol Cheloveka.

[10] Soroko SI, Trubachev VV (2010). Neurophysiological and psychophysiological fundamentals of adaptive bioregulation.

[11] Task Force of the European Society of Cardiology and the American Society of Pacing and Electrophysiology (1996). Heart rate variability: standards of measurement, physiological interpretation, and clinical use.. Circulation.

[12] Muskulus M, Verduyn-Lunel S (2011). Wasserstein distances in the analysis of time series and dynamical systems.. Physica D.

[13] Agelink MW, Boz C, Ullrich H, Andrich J (2002). Relationship between major depression and heart rate variability. Clinical consequences and implications for antidepressive treatment.. Psychiatry Res.

[14] Schmidt H, Muller-Werdan U, Hoffmann T, Francis DP, Piepoli MF (2005). Autonomic dysfunction predicts mortality in patients with multiple organ dysfunction syndrome of different age groups.. Critical Care Medicine.

[15] Romanowicz M, Schmidt JE, Bostwick JM, Mrazek DA, Karpyak VM (2011). Changes in heart rate variability associated with acute alcohol consumption: current knowledge and implications for practice and research.. Alcohol Clin Exp Res.

[16] Mezic I, Runolfsson T (2008). Uncertainty propagation in dynamical systems.. Automatica.

[17] Ni K, Bresson X, Chan T, Esedoglu S (2009). Local histogram based segmentation using the Wasserstein Distance.. International Journal of Computer Vision.

[18] Malliani A, Julien C, Billman GE, Cerutti S, Piepoli MF (2006). Cardiovascular variability is/is not an index of autonomic control of circulation: point/counterpoint comments.. Journal of Applied Physiology.

[19] Malpas SC (2002). Neural influences on cardiovascular variability: possibilities and pitfalls.. American Journal of Physiology: Heart and Circulatory Physiology.

[20] Goldstein DS, Bentho O, Park MY, Sharabi Y (2011). LF power of heart rate variability is not a measure of cardiac sympathetic tone but may be a measure of modulation of cardiac autonomic outflows by baroreflexes.. Exp Physiol., ePub ahead of print.

[21] Vaschillo E, Lehrer P, Rishe N, Konstantinov M (2002). Heart rate variability biofeedback as a method for assessing baroreflex function: a preliminary study of resonance in the cardiovascular system.. Applied Psychophysiology and Biofeedback.

[22] Vaschillo EG, Vaschillo B, Buckman JF, Pandina RJ, Bates ME, Fred A, Filipe J, Gamboa H (2011). The investigation and clinical significance of resonance in the heart rate and vascular tone baroreflexes.. Biomedical Engineering Systems and Technologies: Communications in Computer and Information Science.

[23] Aljuri N, Cohen RJ (2004). Theoretical considerations in the dynamic closed-loop baroreflex and autoregulatory control of total peripheral resistance.. American Journal of Physiology: Heart and Circulatory Physiology.

[24] Yasumasu T, Reyes Del Paso GA, Takahara K, Nakashima Y (2006). Reduced baroreflex cardiac sensitivity predicts increased cognitive performance.. Psychophysiology.

[25] Liao D, Barnes RW, Chambless LE, Simpson RJ, Sorlie P (1995). Age, race, and sex differences in autonomic cardiac function measured by spectral analysis of heart rate variability–the ARIC study. Atherosclerosis Risk in Communities.. Am J Cardiol.

[26] Evans JM, Ziegler MG, Patwardhan AR, Ott JB, Kim CS (2001). Gender differences in autonomic cardiovascular regulation: spectral, hormonal, and hemodynamic indexes.. Journal of Applied Physiology.

[27] Li Z, Snieder H, Su S, Ding X, Thayer JF (2009). A longitudinal study in youth of heart rate variability at rest and in response to stress.. International Journal of Psychophysiology.

[28] Udo T, Bates ME, Mun EY, Vaschillo EG, Vaschillo B (2009). Gender differences in acute alcohol effects on self-regulation of arousal in response to emotional and alcohol-related picture cues.. Psychology of Addictive Behaviors.

[29] Rossy LA, Thayer JF (1998). Fitness and gender-related differences in heart period variability.. Psychosomatic Medicine.

[30] Kraemer S (2000). The fragile male.. British Medical Journal.

[31] Barford A, Dorling D, Davey Smith G, Shaw M (2006). Life expectancy: women now on top everywhere.. BMJ.

[32] Vaschillo EG, Bates ME, Vaschillo B, Lehrer P, Udo T (2008). Heart rate variability response to alcohol, placebo, and emotional picture cue challenges: effects of 0.1-Hz stimulation.. Psychophysiology.

[33] Labiner DM, Ahern GL (2007). Vagus nerve stimulation therapy in depression and epilepsy: therapeutic parameter settings.. Acta Neurol Scand.

[34] Lehrer PM, Vaschillo E, Vaschillo B (2000). Resonant frequency biofeedback training to increase cardiac variability: rationale and manual for training.. Applied Psychophysiology and Biofeedback.

[35] Beekwilder JP, Beems T (2010). Overview of the clinical applications of vagus nerve stimulation.. J Clin Neurophysiol.

[36] Karavidas MK, Lehrer PM, Vaschillo E, Vaschillo B, Marin H (2007). Preliminary results of an open label study of heart rate variability biofeedback for the treatment of major depression.. Applied Psychophysiology and Biofeedback.

[37] Lehrer PM, Vaschillo E, Vaschillo B, Lu SE, Scardella A (2004). Biofeedback treatment for asthma.. Chest.

[38] Jennings JR, Kamarck T, Stewart C, Eddy M, Johnson P (1992). Alternate cardiovascular baseline assessment techniques: vanilla or resting baseline.. Psychophysiology.

[39] Lang PJ, Bradley MM, Cuthbert BN (1999). International affective picture system (IAPS): instruction manual and affective ratings..

[40] Battig WF, Montague WE (1969). Category norms for verbal items in 56 categories: a replication and extension of the Connecticut category norms.. Journal of Experimental Psychology Monograph.

[41] Siegle GJ (1994). The balanced affective word list creation program.. http://www.sci.sdsu.edu/CAL/wordlist/.

